# Absolute quantification of cerebral tissue oxygen saturation with multidistance broadband NIRS in newborn brain

**DOI:** 10.1364/BOE.412088

**Published:** 2021-01-15

**Authors:** Zuzana Kovacsova, Gemma Bale, Subhabrata Mitra, Frédéric Lange, Ilias Tachtsidis

**Affiliations:** 1 Department of Medical Physics & Biomedical Engineering, University College London, London, WC1E 6BT, UK; 2 Department of Engineering, University of Cambridge, Cambridge, CB2 1PZ, UK; 3 Department of Physics, University of Cambridge, Cambridge, CB3 0HE, UK; 4 Institute for Women’s Health, University College London and Neonatal Unit, University College London Hospitals Trust, London, NW1 2BU, UK

## Abstract

Tissue oximetry with near-infrared spectroscopy (NIRS) is a technique for the measurement of absolute tissue oxygen saturation (StO_2_). Offering a real-time and non-invasive assessment of brain oxygenation and haemodynamics, StO_2_ has potential to be used for the assessment of newborn brain injury. Multiple algorithms have been developed to measure StO_2_, however, issues with low measurement accuracy or extracranial tissue signal contamination remain. In this work, we present a novel algorithm to recover StO_2_ in the neonate, broadband multidistance oximetry (BRUNO), based on a measurement of the gradient of attenuation against distance measured with broadband NIRS. The performance of the algorithm was compared to two other published algorithms, broadband fitting (BF) and spatially resolved spectroscopy (SRS). The median error when recovering StO_2_ in light transport simulations on a neonatal head mesh was 0.4% with BRUNO, 4.2% with BF and 9.5% with SRS. BRUNO was more sensitive to brain tissue oxygenation changes, shown in layered head model simulations. Comparison of algorithm performance during full oxygenation-deoxygenation cycles in a homogeneous dynamic blood phantom showed significant differences in the dynamic range of the algorithms; BRUNO recovered StO_2_ over 0–100%, BF over 0–90% and SRS over 39–80%. Recovering StO_2_ from data collected in a neonate treated at the neonatal intensive care showed different baseline values; mean StO_2_ was 64.9% with BRUNO, 67.2% with BF and 73.2% with SRS. These findings highlight the effect of StO_2_ algorithm selection on oxygenation recovery; applying BRUNO in the clinical care setting could reveal further insight into complex haemodynamic processes occurring during neonatal brain injury.

## 1. Introduction

Tissue oximetry with near-infrared spectroscopy (NIRS) is a non-invasive technique for assessing tissue oxygenation and haemodynamics through the measurement of tissue oxygen saturation StO_2_. StO_2_ is defined as the ratio of the concentration of oxygenated haemoglobin c_HbO2_ to total haemoglobin concentration (c_THb_ = c_HbO2_ + c_HHb_). The absorption of light in tissue in the near-infrared region (650–900nm) is dominated by oxygenated haemoglobin (HbO_2_) and deoxygenated haemoglobin (HHb), whose concentrations can be quantified using NIRS. As haemoglobin is the main oxygen-carrier in blood, measuring tissue oxygen saturation informs on the balance between oxygen supply and demand.

Cerebral tissue oximetry can be used in various settings in the hospital including the intensive care unit, during cardiac surgery, anaesthesia or in brain injury management. One area of significant interest is the use in the neonatal intensive care (NICU) for assessment of neonatal brain injury in term [1] or in preterm infants [2]. However, a 2018 worldwide survey conducted across 235 NICUs showed that only 36% owned a NIRS device and the majority did not use it regularly [3]. The reasons for not using the technique were concerns about the current evidence for financial and clinical benefits. Despite the ongoing research efforts to demonstrate benefits of tissue oximetry in neonatal intensive care, including clinical trials like SafeBooosC [2], a lack of consistency among instrumentation makes associating tissue oximetry with clinical outcomes challenging [4].

The lack of clinical evidence is also hindered by the presence of various issues which have faced brain oximetry since its introduction and need to be addressed. These include:

1. Extracerebral signal contamination: the presence of signal components originating in the extracerebral layer. Superficial layer contamination is weaker in neonates than in adults due to their thin skulls so StO_2_ tracks the oxygenation of brain tissue [5,6]. The impact of extracerebral tissue on StO_2_ can also be decreased through the introduction of multidistance measurements [7] or algorithms using two-layer models of light transport in tissue [8]. Nevertheless, it has been shown that even with the use of multidistance measurement setups, changes in scalp tissue oxygenation do affect the accuracy of cerebral oximeters [9,10].
2. Validation: the ability of oximeters to detect true alterations in the oxygenation state of a tissue is quantified by the accuracy, measured through the comparison to a gold standard. This gold standard is often an invasive measurement of blood oxygenation in subjects and is calculated assuming a fixed ratio of venous to arterial blood. However, it is not possible to quantify how much each vascular compartment contributes to the overall light attenuation. StO_2_ is burdened by the contribution of extracerebral layers and its *in vivo* validation is not feasible in vulnerable subject groups, such as preterm infants [11]. An alternative to *in vivo* validation is the use of optical phantoms [12] but it is impossible to perfectly mimic tissue physiology in such an environment.
3. Precision: precision is a measure of the agreement between repeated measurements with a single device. Treatment decisions in clinical practice are often based on single measurements so high precision is desired, a reasonable value to aim for is 2–3%, corresponding to spontaneous variability over time in stable patients [13]. Recently, precision of the OxyPrem v1.3 oximeter was reported below 1.85% in neonates [14] after reducing the contribution of spontaneous systemic haemodynamic fluctuations caused by processes including changes in cerebral blood flow or oxygen consumption due to different regulation processes. However, studies conducted using other oximeters mostly report precision unsuitable for clinical use [15–17].
4. Accuracy and disagreement between different oximeter devices: one cause for the difficulty in establishing reliable intervention thresholds for various patient groups are differences in oximeter readings. The comparison of the performance of multiple oximeters reports disagreement in human studies [18–20] and in phantom studies [12,21]. Differences were found in the dynamic range of instruments [22], in the absolute changes in StO_2_ [23] and their response time [24]. It has also been shown that oximeters can measure a physiologically plausible StO_2_ value despite improper attenuation data collection [25], highlighting the need for quality assessment of the input data prior to analysis.

It is evident that further research is needed to establish the clinical efficacy of oximetry in the clinical care. The focus should be on establishing standardisation protocols in study design and in further developing the instrumentation and data analysis in oximetry to obtain precise and accurate measurements.

Our aim is to improve the performance of oximetry through the development of a novel algorithm to calculate StO_2_ with continuous-wave (CW) NIRS in the neonate. The objective is to have a measure of StO_2_ sensitive to cerebral tissue, that tracks oxygenation with high accuracy, dynamic range and precision, and to include an automated data-quality assessment step in the analysis pipeline to ensure optimal oxygenation assessment. The motivation is to improve cerebral monitoring in infants with hypoxic-ischaemic encephalopathy (HIE), which is a neurological pathology following perinatal hypoxic ischaemic injury. A recent systematic review [26] suggested that monitoring cerebral oximetry might be helpful in HIE, but it is still difficult to draw firm conclusions on the prognostic value of StO_2_ in this group, particularly on individual basis. In this work, we investigate the influence of algorithm selection on StO_2_ measurement and introduce a novel oximetry algorithm, BRoadband mUltidistaNce Oximetry (BRUNO). BRUNO is a hybrid algorithm based on two other oximetry algorithms; a broadband approach based on differential spectroscopy, further referred to as ‘broadband fitting’ (BF), and spatially resolved spectroscopy (SRS), a multidistance approach relying on a measurement of the attenuation slope to quantify scaled absorption and then StO_2_. BF [27] has been evaluated in Monte Carlo studies and compared to time-resolved (TR) NIRS measurements [28]; it has also been applied in preterm infants [29]. SRS, on the other hand, has been developed in the mid-1990s [30] and is used commercially in NIRO devices (Hamamatsu Photonics, Japan) [31] and in research instruments [32,33].

The accuracy of all three algorithms was assessed using two types of NIRS data; broadband spectra obtained in computational simulations, and data collected with a broadband multidistance system in a dynamic optical phantom. A reference oxygenation measurement in the phantom was collected simultaneously with a time resolved NIRS system. Finally, we demonstrate the application of BRUNO in the NICU on data collected in an infant with HIE.

## 2. Methods

### 2.1 Instrumentation

CW NIRS spectral data was collected with an in-house built broadband multidistance system, CYRIL [34]. The system was design specifically for neonatal applications. CYRIL is described in [34], with minor changes to the setup including a more compact light source HL-2000-HP (Ocean Optics, USA) and recalibration to a wider spectral bandwidth, 704–911 nm. The system benefits from a CCD camera with a two-dimensional array of 512×512 pixels, allowing the detection of light from 8 detectors simultaneously. The detector fibres were held in place with a custom-made probe holder. The standard setup is the use of 4 detectors for one measurement channel with the light source-detector separations (SDS) of 15, 20, 25 and 30 mm. Exposure of the system was set to 1 s, data were collected at a rate of 0.9 Hz.

TR-NIRS data were collected with an in-house developed system, named MAESTROS [35]. Briefly, MAESTROS is based on a supercontinuum laser coupled with an acousto-optical tunable filter that permits the selection of 16 narrow wavelengths, with a full width at half maximum between 2 and 4 nm, in the range of 650 to 1100 nm. The light is then transmitted to the tissue via a single core optical fibre. The system allows up to two sources points. On the detection side, four optical fibres collect the reflected light to four photon multiplier tubes. Then, a router is used to redirect the signal to a single time correlated single photon counting card, in order to measure the distribution of the arrival time of the photons (DTOFs). In this work, only one channel with a 30 mm SDS was used. Moreover, the 16 wavelengths used were from 780 to 870 nm, in steps of 6 nm, with an acquisition frequency of 0.5 Hz for one set of 16 wavelengths.

### 2.2 NIRFAST simulations

NIRFAST [36,37], an open-source software toolbox for MATLAB (MathWorks, USA), was used to simulate broadband multidistance NIRS measurements in a neonatal head. We generated two types of data from a neonatal head mesh model; three meshes with static optical properties, and a mesh with optical properties changed in graduated steps. The oxygenation in brain tissue and in the extracerebral layer was changed to evaluate the sensitivity to brain oxygenation and the impact of scalp oxygenation on the StO_2_ signal.

The neonatal model was a segmented mesh built from averaging MRI images of neonates of 40-week gestational age [38]. The mesh consisted of 6 layers; extracerebral tissue (ECT), cerebrospinal fluid (CSF), grey matter (GM), white matter (WM), cerebellum and brainstem. Light sources were placed on the forehead to simulate a measurement with CYRIL, and data collected at 15, 20, 25 and 30 mm SDS were analyzed. The optical properties were set according to [39,40], the three models with constant optical properties are described in Table 1. The properties of the cerebellum and brainstem were always equal to WM. Water concentration was set to 0% in models 1 and 2 to assess the ability of the algorithms to accurately distinguish the absorption by HHb and HbO_2_ without any additional absorption by water, unaccounted for by SRS. Random noise with 1% maximal intensity amplitude was added to each simulated spectrum, generating 50 different spectra for each model.

**Table 1.** Optical properties of a homogeneous and multi-layered neonatal head mesh set in three different NIRFAST simulation models. WF is water fraction, c_HbO2_ is HbO_2_ concentration, c_HHb_ is HHb concentration, a and b describe the wavelength dependence of scattering; µ_s_’ = a(λ/1000)^-b^.

		WF	c_HbO2_	c_HHb_	a	b	StO_2_
Model	Layer	[%]	[µM]	[µM]	[mm^-1^]	[-]	[%]
1	all	0	55.0	29.5	0.5	1.7	65.1

2	ECT	0	43.8	15.7	0.7	0.9	73.6
	CSF	0	0	0	0.3	∼ 0	0
	GM	0	55.0	29.5	0.5	1.7	65.1
	WM	0	67.0	20.9	0.8	1.3	76.2

3	ECT	30	48.2	17.3	0.7	0.8	73.6
	CSF	99	0	0	0.3	∼ 0	0
	GM	80	60.5	32.5	0.5	1.6	65.1
	WM	80	73.7	23.0	0.7	1.2	76.2

Optical properties of brain tissue and ECT were changed in model 4. The mesh was used as a two-layer model, with an extracerebral (ECT, CSF) and a brain layer (GM, WM). The oxygenation of both layers was changed in 23 steps - the oxygenation of each layer was changed between 10% and 90% while the other layer remained constant, and then the oxygenation was changed in both layers simultaneously. The baseline optical properties are described in Table 2, taken from [41]. Total haemoglobin concentration c_THb_ remained constant. Random noise with 1% maximal intensity amplitude was added to each spectrum.

**Table 2.** Optical properties of a two-layer neonatal head mesh set in a series of ECT and brain tissue oxygenation change simulations, model 4.

	c_THb_	a	b	StO_2_
Layer	[µM]	[mm^-1^]	[-]	[%]
ECT	59.5	1.6	1.1	10–90
Brain	76.0	0.8	1.6	10–90

### 2.3 Phantom measurements

A homogeneous dynamic liquid blood phantom was used to collect broadband multidistance data with CYRIL simultaneously with MAESTROS to obtain a reference StO_2_ measurement. A detailed description of the phantom setup can be found in [35]. The liquid was a mixture of water, phosphate buffer, intralipid, blood and yeast. The phantom was oxygenated and deoxygenated in cycles; the liquid was continuously stirred with a magnetic stirrer and the temperature was kept at 37 ± 1 °C. Oxygen from a gas tank was delivered through an air stone to oxygenate the mixture; the mixture was deoxygenated by yeast consuming the oxygen. The oxygen content of the mixture was monitored with a diffuse oxygen content probe. When the diffused oxygen content of the mixture dropped to 0%, the mixture was fully deoxygenated, maximal oxygenation of the liquid led to the diffuse oxygen probe plateauing at values above 100%. The protocol of the measurement is in Table 3.

**Table 3.** Liquid phantom measurement protocol

Step	
1	Baseline measurement
2	Add 15 g of yeast
3	Wait for deoxygenation plateau
4	Turn on oxygen supply
5	Wait for oxygenation plateau
6	Turn off oxygen supply
7	Repeat steps 3 to 6 multiple times
8	End

The surface area of the phantom was large enough for the use of two NIRS systems without interference. Optical fibres were held in place by custom probe holders placed on the surface of the liquid and slightly submerged to ensure no gas bubbles would be trapped underneath the optodes. CYRIL data were collected with one channel only, with 4 detectors at the standard SDS of 15, 20, 25 and 30 mm.

### 2.4 Measurement in a newborn with HIE

Monitoring of a term infant with HIE with CYRIL was performed during the first days of life as part of the UCLH Baby Brain Study; approved by the NW London Research Ethics Committee 2 (Reference 13/LO/0225). Written, informed consent was obtained from parents before the study. The infant was treated with therapeutic hypothermia, magnetic resonance spectroscopy (MRS) was performed after completion of rewarming. NIRS measurement with CYRIL was commenced as early as possible. The study was conducted while the infant was monitored with EEG; CYRIL optodes were placed on the forehead without disturbing the EEG electrodes. The CYRIL setup comprised of 4 detectors on each side of a central light source with SDS of 15, 20, 25 and 30 mm; CYRIL collected data from two measurement channels, with 4 detectors per hemisphere. The probe holder was attached to the skin using clinically safe double-sided tape. The probes were tucked underneath the EEG cap.

### 2.5 Data analysis

**Fig. 1.**
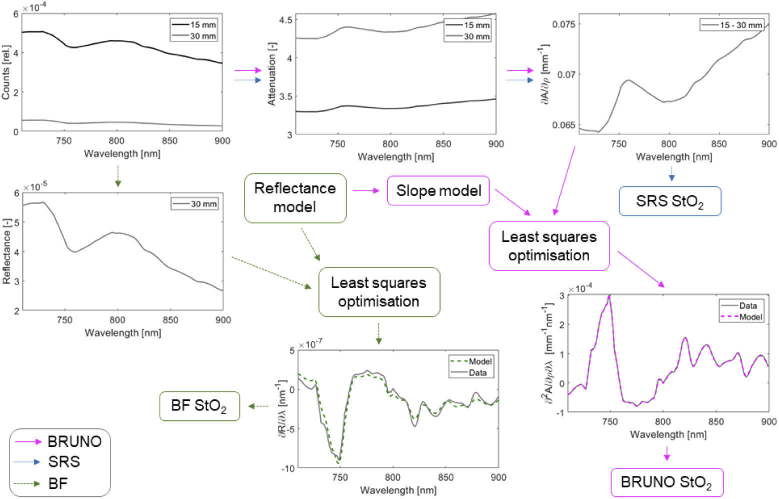
Diagram showing the application of broadband multidistance oximetry (BRUNO), broadband fitting (BF) and spatially resolved spectroscopy (SRS) to a broadband spectrum obtained in NIRFAST model 3. ∂A/∂ρ is the slope of attenuation against distance, ∂^2^A/∂ρ∂λ is the spectral derivative of the attenuation slope, ∂R/∂λ is the spectral derivative of reflectance. In BF, both the first and second spectral derivatives of reflectance are used but only the first is shown in this diagram.

Broadband spectra were analysed with three different algorithms to obtain StO_2_; SRS, BF and BRUNO. All analysis was performed in MATLAB version 2019b (Mathworks, Natick, USA). The diagram in Fig. 1 shows the steps in applying all three algorithms to a broadband spectrum obtained in NIRFAST model 3, leading to StO_2_.

#### 2.5.1 Broadband fitting

BF is based on a single-distance measurement of reflectance, R, to which the solution to the diffusion approximation for a semi-infinite homogeneous medium, R_t_, calculated with extrapolated boundary conditions (EBC), is applied [42]. As a single-distance measurement can lead to a significant contribution of extracerebral signal contamination [5], the algorithm is not suited for measurements in adults due to their thicker skulls but is suitable for measurements in neonates where the contribution of skull thickness to the measurement error is smaller [5,6]. The fitting of R_t_ to R was achieved through three steps. First, the measured reflectance was smoothed with a 5-point moving average filter, then the reflectance spectra were twice differentiated with respect to wavelength and, lastly, the reflectance derivatives were fitted to the numerical derivatives of the theoretical model. BF was applied across the range of 710 nm to 850 nm. The fitting of the theoretical model to the measured reflectance was achieved with least-squares optimization *fminsearchbnd* [43]. The optimization converged after an iterative search of the absorption coefficient µ_a_ and the transport scattering coefficient µ_s_’,

(1) $$\mu_{a}(\lambda )=c_{HbO_{2}}\alpha_{HbO_{2}}(\lambda )+c_{HHb}\alpha_{HHb}(\lambda )+WF\mu_{a_{H_{2}O}}(\lambda )$$

(2) $${\mu_{s}}^{\prime }(\lambda )=a{\left(\frac{\lambda }{1000}\right)}^{-b}$$

where λ is wavelength, α is the specific absorption coefficient, WF is the water fraction, c_HbO2_ and c_HHb_ are the concentrations of HbO_2_ and HHb, a is scattering amplitude and b is scattering power. Finding c_HbO2_ and c_HHb_ led to StO_2_:

(3) $$StO_{2}=\frac{c_{HbO_{2}}}{c_{HHb}+c_{HbO_{2}}}\times 100\%$$

#### 2.5.2. Spatially resolved spectroscopy

SRS uses a multidistance measurement of attenuation to calculate the slope of attenuation, A, against distance, ρ. Based on the zero-boundary conditions (ZBC) solution to the diffusion equation for a semi-infinite homogeneous medium, the attenuation slope ∂A/∂ρ for detectors spaced apart at separations similar to the shortest SDS (5 mm inter-detector separation, 15 mm shortest SDS for CYRIL) is given by [32]:

(4) $$\frac{\partial A}{\partial \rho }=\frac{1}{\ln(10)}\left(\sqrt{3\mu_{a}{\mu_{s}}^{\prime }}+\frac{2\ln\left(\frac{d_{L}}{d_{S}}\right)}{d_{L}-d_{S}}\right)$$

where d_L_ is the longest SDS and d_S_ is the shortest SDS. As both µ_a_ and µ_s_’ remain unknown, µ_s_’ was simplified through a linear approximation:

(5) $${\mu_{S}}^{\prime }=k(1-h\lambda )$$

where k is an unknown constant and h is the normalized gradient of µ_s_’ against λ and equal to h = 0.00063 mm^-1^nm^-1^ [31]. Substituting Eq. (5) in (Eq. (4)) and measuring A at two wavelengths λ_1_ and λ_2_ yields an expression for a scaled absorption coefficient kµ_a_:

(6) $$k\mu_{a}(\lambda )=\frac{1}{3(1-h\lambda )}\left(\ln(10)\frac{\partial A}{\partial \rho }-\frac{2\ln\left(\frac{d_{L}}{d_{S}}\right)}{d_{L}-d_{S}}\right)$$

The scaled concentrations of HHb and HbO_2_, kc_HHb_ and kc_HbO2_ were found by solving

(7) $$\left(\begin{array}{c} kc_{HHb}\\ kc_{HbO_{2}} \end{array}\right)={\left(\begin{array}{cc} \alpha_{HHb}(\lambda_{1}) & \alpha_{HbO_{2}}(\lambda_{1})\\ \alpha_{HHb}(\lambda_{2}) & \alpha_{HbO_{2}}(\lambda_{2}) \end{array}\right)}^{-1}\left(\begin{array}{c} k\mu_{a}(\lambda_{1})\\ k\mu_{a}(\lambda_{2}) \end{array}\right)$$

StO_2_ was calculated from the scaled concentrations, equivalent to (Eq. (3)). The wavelengths at which SRS is applied varies among instruments, 3 or 4 wavelengths are typically selected in the near-infrared range. In this work, SRS was applied across the whole broadband wavelength range of CYRIL in data collected with all 4 detectors.

#### 2.5.3 Broadband multidistance oximetry

BRUNO was designed as a combination of BF and SRS to exploit the advantages of both methods. Similarly to SRS, the attenuation slope against distance ∂A/∂ρ was measured in a multidistance setup. Instead of approximating µ_s_’ using a constant, both µ_s_’ and µ_a_ were found through the fitting of the measured attenuation slope to the ZBC slope model in Eq. (4). The fitting was performed in the first spectral derivative space in the wavelength range 710–900 nm; the measured slope was first smoothed using a 5-point moving average filter. The optimization routine *fminsearchbnd* [43] then gave the best fit with the recovery of parameters WF, c_HbO2_, c_HHb_, a and b from ∂^2^A/∂ρ∂λ. StO_2_ was calculated from Eq. (3). A MATLAB script to run BRUNO on attenuation slope data is available on GitHub [44]. In this work, the attenuation slope was calculated across all 4 detectors with SDS 15, 20, 25 and 30 mm.

The boundary conditions for *fminsearchbnd* for the analysis of all data with BF and BRUNO are reported in Table 4.

**Table 4.** Boundary conditions for analysis with BRUNO and BF. LB – lower boundary, UB – upper boundary. The boundary conditions for NIRFAST and phantom data were based on the ground truth and on values found in literature for neonatal measurements.^*a*^

		WF ^*b*^	c_HbO2_	c_HHb_	a	b
		[%]	[µM]	[µM]	[mm^-1^]	[-]
NIRFAST	Start	70	50	50	1.0	1.0
	LB	60	0	0	0.3	0.5
	UB	95	100	100	2.0	3.0

	Start	100	20	20	1.0	3.0
Phantom	LB	97	0	0	0.0	0.0
	UB	100	40	40	2.0	4.0

	Start	80	100	40	0.5	0.5
Neonate	LB	50	0	0	0.1	0.1
	UB	95	160	70	2.0	3.0

^*a*^ From Spinelli, L., Zucchelli, L., Contini, D., et al (2017) Neurophotonics, 4(04) [45]

^*b*^ WF was set to 0% for NIRFAST models without any water absorption.

#### 2.5.4 Data quality evaluation

The differentiation step in BF and BRUNO amplifies the noise component of spectra which decreases the visibility of spectral features of chromophores. To ensure that data were suitable for analysis, that the BRUNO algorithm could converge to an accurate solution, an automated data quality assessment step was added to the pipeline.

**Fig. 2.**
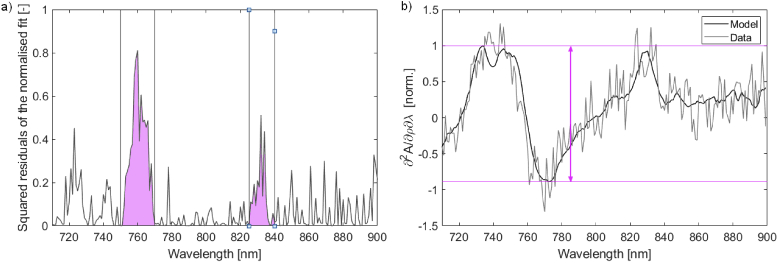
Visualization of the calculation of the evaluation score. (a) Highlighted wavelength regions where the sum of squared residuals is calculated. (b) The pink arrow indicates the range of the fit, the span between the highest and lowest point of the model.

The following process was developed empirically and was based on the visual assessment of the goodness of fit of the theoretical slope model to the measured data. First, BRUNO was used to obtain a theoretical model that best matched the measured slope. The spectral derivatives of both the model and the slope were normalized using the MATLAB function *normalize* and the squared residuals of the fit were calculated. Then, the sums of squared residuals between 750–770 nm and between 825–840 nm were multiplied, obtaining ∏R. The selection of the ranges where residuals were calculated is shown in Fig. 2(a).

The second part of the evaluation scoring was based on the range of the model, r. The range was defined as the difference between the minimum and maximum of the model, shown in Fig. 2(b). Introducing a range measurement penalised spectra that did not have distinct spectral features. The final score was calculated by multiplying the product of the two residual sums with the reciprocal of the range, score = ∏R × 1/r. The lower the score, the higher the data quality was. Scoring different data sets collected in the phantom and neonate and visually assessing the quality of the fit led to a threshold value of 2; spectra with a score lower than this threshold were suitable for analysis.

#### 2.5.5 MAESTROS data analysis

The data analysis for the MAESTROS system was the same as reported in [35]. Briefly, the DTOFs were fitted using a standard model of diffusion theory [46] after convolution with the IRF. We then calculated the absolute concentrations c_HbO2_ and c_HHb_ (assuming a water concentration of 98%), using the Beer-Lambert law. Finally, c_HbO2_ and c_HHb_ were then used to calculate the absolute StO_2_ using Eq. (3).

#### 2.5.6 Statistical analysis

The median StO_2_ was calculated for NIRFAST simulations from models 1, 2 and 3. The agreement between the recovered StO_2_ with either algorithm and the oxygenation of the brain layer in model 4 was visualised using a scatter plot, generated using the MATLAB package Bland-Altman and Correlation Plot [47]. Multilinear regression was used to quantify the influence of brain oxygenation and ECT oxygenation on the recovered StO_2_ in model 4, based on the equation StO_2_ = constant + x_1_Brain + x_2_ECT. The scale of x_2_ indicated the influence of ECT oxygenation on the recovered StO_2_; if the recovered StO_2_ was equal to the oxygenation of the brain, the regression equation was StO_2 _= 0 + 1×Brain + 0×ECT. Scatter plots were also used to compare the results obtained with BRUNO, SRS and BF to the measurements with MAESTROS, correlation was assessed using Spearman’s rank correlation, ρ_s_.

## 3. Results

### 3.1. NIRFAST simulations

**Fig. 3.**
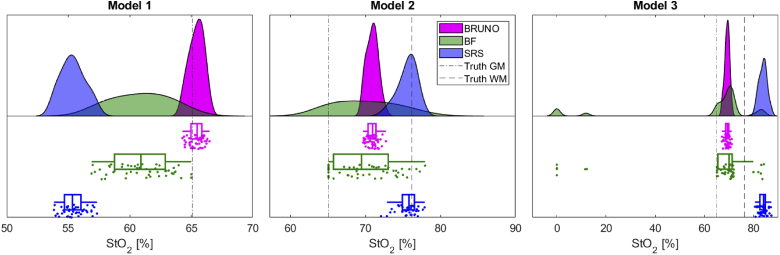
StO_2_ recovered in 3 different NIRFAST models with 50 spectra analysed each. Model 1 was a homogeneous model with grey matter (GM) optical properties, without water absorption, model 2 was a heterogeneous model without water absorption and model 3 was a heterogeneous model with water absorption. The true oxygenations of grey matter (GM) and white matter (WM) are indicated with lines. The figure was generated using the Raincloud plots toolbox [48].

The StO_2_ recovered with BRUNO, BF and SRS from spectra simulated in models 1, 2 and 3 is shown in Fig. 3. The true oxygenations of GM (65.1%) and WM (76.2%) are indicated. Model 1 was homogeneous; the true oxygenation was equal to GM; models 2 and 3 were heterogeneous and the truth was expected to be an average of GM and WM oxygenation.

The differences in the algorithm performances lie in the accuracy and the width of the distribution of the results, reported in Table 5. BRUNO recovered StO_2_ with the highest accuracy in model 1 and recovered an StO_2_ which was a combination of GM and WM oxygenation in models 2 and 3. SRS was affected the least by the addition of random noise; the distribution of the results was very narrow. The accuracy of the SRS algorithm was the lowest; it underestimated oxygenation in model 1 and overestimated it in models 2 and 3. BF was accurate in models 2 and 3 and slightly underestimated StO_2_ in model 1. The distribution of BF results was the widest.

**Table 5.** Median oxygenation StO_2_ recovered with BRUNO, BF and SRS in NIRFAST models 1, 2 and 3. The 25^th^ and 75^th^ percentiles are also reported.

	Median StO_2_ (25^th^–75^th^ percentile) [%]		
Algorithm / Model	1	2	3
BRUNO	65.5 (65.0–65.9)	71.0 (70.4–71.4)	69.4 (68.7–69.9)
BF	60.9 (58.8–62.9)	69.5 (65.6–73.1)	70.0 (65.5–71.3)
SRS	55.6 (55.6–55.6)	75.8 (75.8–75.9)	84.4 (84.3–84.4)

**Fig. 4.**
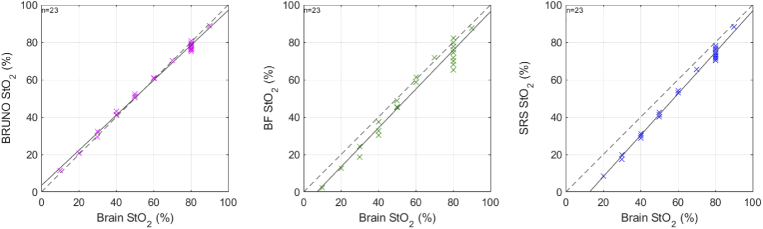
Scatter plot of the recovered StO_2_ versus the true oxygenation of the inner layer, brain, during oxygenation changes in the ECT and the brain layer simulated in NIRFAST. Multiple values of StO_2_ at different baseline oxygenations correspond to different ECT oxygenations. The dashed line is the identity line, the full line is the regression line.

The ability of the three algorithms to track oxygenation during oxygenation changes in a two-layer model, model 4, is shown in scatter plots in Fig. 4. The recovered StO_2_ was plotted against the true oxygenation of the brain layer. Multiple StO_2_ values were recovered for some true oxygenation values as they correspond to different ECT layer oxygenations.

Based on the scatter plot in Fig. 4, BRUNO followed the oxygenation of brain more closely than SRS and BF, with the regression line overlapping with the identity line. Both BF and SRS underestimated oxygenation with an offset which increased with the decrease of brain oxygenation. The accuracy of the recovered StO_2_ to the oxygenation of brain tissue is quantified in Table 6, where the coefficients obtained in multilinear regression of the recovered oxygenation against the oxygenation of brain and ECT. The coefficient x_1_ was closest to 1 in BRUNO and x_2_ was the smallest, suggesting minimal influence of ECT oxygenation on BRUNO StO_2_.

**Table 6.** The coefficients of multilinear regression StO_2_ = constant + x_1_Brain + x_2_ECT performed on the StO_2_ recovered with each algorithm in model 4.

Algorithm	constant	x_1_	x_2_
BRUNO	-2.74	0.96	0.07
BF	-25.53	1.11	0.21
SRS	-22.30	1.14	0.10

### 3.2. Phantom measurements

**Fig. 5.**
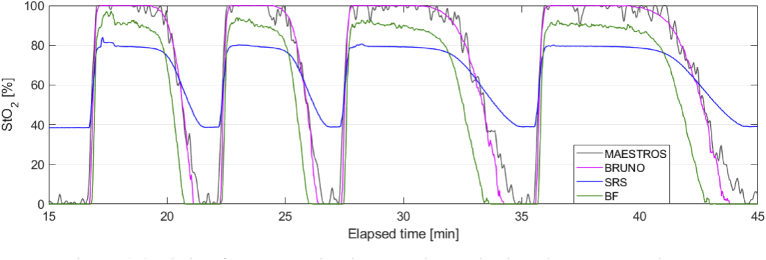
StO_2_ during four oxygenation-deoxygenation cycles in a homogeneous phantom calculated with BRUNO, BF and SRS compared to the reference measurement with MAESTROS.

**Fig. 6.**
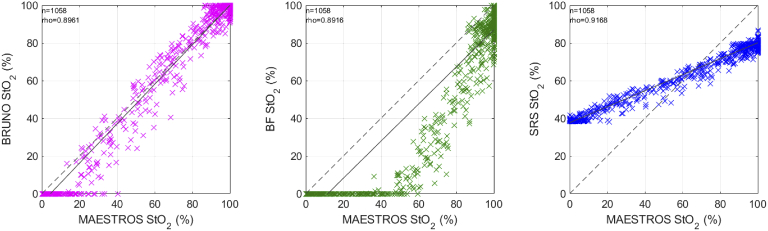
Scatter plot of the recovered StO_2_ versus the reference oxygenation measurement with MAESTROS during oxygenation changes in a homogeneous optical blood phantom. The dashed line is the identity line, the full line is the regression line. rho is Spearman’s rank coefficient.

The quality of phantom data collected with CYRIL was suitable for BRUNO analysis as the data evaluation score did not exceed 2, the mean score was 0.19. StO_2_ calculated with BRUNO, BF and SRS from data collected with CYRIL was compared to the reference measurement of oxygenation with MAESTROS. Figure 5 shows the time series of oxygenation during four oxygenation-deoxygenation cycles. All responses were smoothed with a 5-step moving average filter prior to plotting. Agreement between the three CW algorithms and the MAESTROS baseline was visualised in scatter plots in Fig. 6 where also the correlation coefficient ρ_s_ (rho) is shown. The correlation between MAESTROS and BRUNO was ρ_s _= 0.90, between MAESTROS and BF ρ_s _= 0.90 and between MAESTROS and SRS ρ_s _= 0.92.

Figure 5 shows that agreement between MAESTROS and BRUNO was the highest out of the three algorithms. BRUNO accurately tracked the absolute oxygenation value with the same dynamic range as MAESTROS, 0–100%.

SRS responded to oxygenation changes with almost the same temporal dynamics as MAESTROS, indicated by the highest ρ_s_, but with a reduced dynamic range shown by the tilt of the regression line in respect to the identity line in Fig. 6. StO_2_ did not reach values below 39% and plateaued at 80% during oxygenation.

The agreement between MAESTROS and BF was the lowest; the dynamic range of BF was limited at the maximum saturation (it peaked at ∼90%). BF and MAESTROS changed at a similar rate, but BF responded to increases in oxygenation up to 10 s later than MAESTROS and detected deoxygenation up to one minute earlier than MAESTROS.

The duration of the analysis with each of the three CW algorithms also varied: BRUNO analyzed all 2368 spectra in 62.4 s (26 ms per spectrum), SRS in 1.2 s (0.5 ms per spectrum) and BF in 188.6 s (80 ms per spectrum), on a PC with 32 GB RAM and an Intel Core i7-8700 processor. The duration excludes data loading and pre-processing.

### 3.3. Measurement in a newborn with HIE

We present data collected in one infant. The patient was female, gestational age 40 weeks + 5 days, birth weight 2.3 kg. MRS after rewarming reported no abnormalities; the neonate was diagnosed with moderate HIE. The NIRS data was collected on the third day of life, while the infant was sedated and undergoing therapeutic hypothermia.

Two data channels were collected in the infant simultaneously, from one hemisphere each. The data evaluation score was calculated for each channel. The mean score of measurements on the left side was 1.3, the mean score of measurements on the right side was 10.4, indicating poor quality spectra on the right side.

**Fig. 7.**
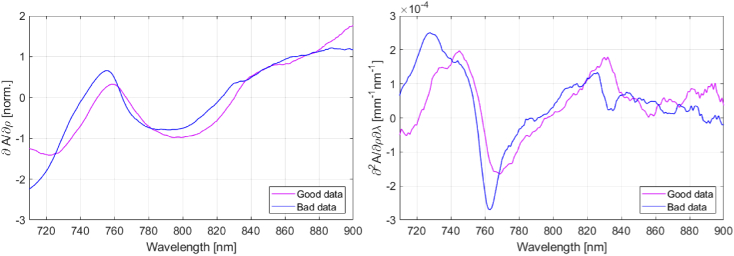
Attenuation slope ∂A/∂ρ and its spectral differential ∂^2^A/∂ρ∂λ collected with two different measurement channels. The suitable data (‘good data’) was collected on the left side of the infant’s head (mean score 1.3), the unsuitable data (‘bad data’) on the right side (mean score 10.4).

Examples of attenuation slopes and their spectral derivatives collected on either side of the head are shown in Fig. 7. The attenuation slopes were normalised using the MATLAB function *normalize* so that their shapes can be compared and smoothed using a 10-step moving average filter. The spectral derivatives were also smoothed with a 10-step moving average filter. The differentiation highlighted the differences in the two spectra; particularly the absence of the 830 nm water peak in the ‘bad’ right side spectrum. Based on these findings and the evaluation score, data from the right channel were excluded from the analysis.

Data from the left side were analysed; the mean StO_2_ (standard deviation) measured was 64.9% (4.1%) with BRUNO, 67.2% (2.6%) with BF and 73.2% (2.2%) with SRS, while mean WF was 87.9% (10.8% standard deviation) recovered with BRUNO.

## 4. Discussion

A novel algorithm to recover StO_2_ from broadband, multidistance CW NIRS data is presented. The algorithm was applied to three types of NIRS data; spectra obtained in NIRFAST simulations in a layered neonatal head mesh, measurements in a dynamic optical phantom and a neonate with HIE treated at the NICU.

BRUNO was developed with the aim to be an oximetry algorithm based on multidistance broadband NIRS, which would recover StO_2_ with high accuracy, a high dynamic range and precision, and show minimal signal contamination from extracerebral layers. We have shown that BRUNO has recovered StO_2_ with higher accuracy than SRS and BF in simulated and phantom data, that it can track oxygenation changes in the brain with minimal ECT contamination, and that it has a larger dynamic range than other algorithms.

BRUNO is a hybrid algorithm based on SRS and BF. We have shown that combining the strengths of each algorithm can lead to higher accuracy of the recovered StO_2_ than achievable with either algorithm individually. Table 7 shows a summary of all three algorithms.

**Table 7.** Summary of spatially resolved spectroscopy (SRS), broadband fitting (BF) and broadband multidistance oximetry (BRUNO). ZBC: zero boundary conditions, EBC: extrapolated boundary conditions, MD: multidistance, SD: single-distance.

	SRS	BF	BRUNO
Diffusion equation boundary conditions	ZBC	EBC	ZBC
Set up	MD	SD	MD
Accounts for water absorption	✗	✓	✓
Broadband spectrum	✗ ^*a*^	✓	✓
Low computational requirements	✓	✗	∼
Lower noise amplification	✓	✗	✗
No prior scattering assumption	✗	✓	✓
Reduction of superficial layer contamination	✓	✗	✓

^*a*^ A broadband measurement is not necessary for the use of SRS but is possible.

**Fig. 8.**
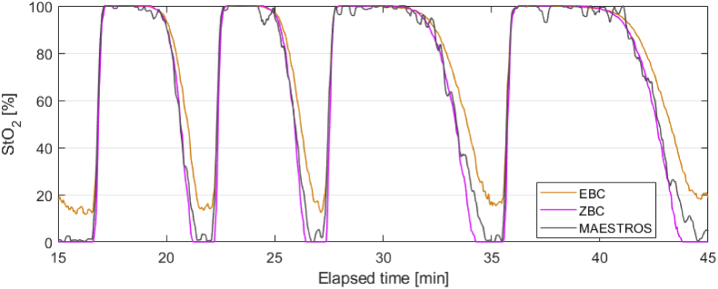
Comparison of StO_2_ recovered with BRUNO using either ZBC or EBC compared to MAESTROS. The StO_2_ trace was smoothed with a moving average filter.

While all three algorithms are based on a homogeneous slab tissue model, they differ in the selection of boundary conditions. Using EBC to solve the diffusion equation leads to a more precise approximation of light transport in tissue but using ZBC is sufficient, only introducing large errors close to the light source [42,49,50]. Using EBC was tested during the development of BRUNO but did not lead to an increase of the accuracy of StO_2_ recovery and significantly increased the computational burden of the algorithm. Figure 8. in Appendix shows a comparison of StO_2_ in the homogeneous phantom recovered with BRUNO using both EBC and ZBC models, compared to MAESTROS.

Absorption by water is usually not accounted for in SRS; it is expected that it would not lead to significant errors as water absorption is relatively low compared to haemoglobin absorption in the wavelength range standardly used by SRS instruments, such as 735, 810 and 850 nm in the NIRO-200NX (Hamamatsu Photonics K.K., Japan). However, as the absorption by water becomes more significant at longer wavelengths around 900 nm and differentiation of broadband spectra amplifies otherwise indistinguishable spectral features of the chromophore, it can be accounted for with BF and BRUNO. As our application of SRS was applied over a broad wavelength range, the influence of water absorption was more likely. In fact, the addition of water absorption in model 3 decreased the accuracy of SRS recovered oxygenation compared to model 2, suggesting that the absorption by water was not negligible but the algorithm could not account for it and instead overestimated c_HbO2_. It is possible to include water absorption in SRS either by adding a constant water fraction component or by adding water as an absorbing chromophore in Eq. (7). We did not investigate how accounting for water absorption in SRS would improve the accuracy of SRS analysis in this work but have discussed it in our previous work [51]. Another consideration for further evaluation of BRUNO performance could be changing the water content in GM and WM layers in NIRFAST simulations and exploring the effect on the accuracy of StO_2_.

A desirable benefit of BF is the ability to account for scattering changes with time. SRS on the other hand is limited by using the fixed linear approximation, h in Eq. (5), for scattering, which has been measured in a small sample size of adults and can differ between subject groups. Changing h acts as a baseline offset of StO_2_ and hence affects the precision of StO_2_ [51]. The benefit of BRUNO is that while it is based on SRS, incorporating the broadband measurement removes these prior assumptions about tissue scattering.

NIRFAST simulations of oxygenation changes in ECT and brain tissue allowed assessment of the ability of the algorithms to monitor brain tissue saturation without contamination of ECT. The results showed that BRUNO led to a more brain-specific signal than with BF and SRS, Fig. 4. SRS did track the oxygenation of brain with higher brain tissue sensitivity than BF. The higher sensitivity to changes in brain tissue found with BRUNO and SRS than with BF was because of the use of the multidistance setup and in agreement with previous findings where the ability of SRS to track changes in brain tissue with decreased sensitivity to changes in optical properties of ECT has been shown [52,53].

Despite the ability of SRS to track oxygenation changes in brain tissue with only small contamination of the ECT signal, the estimated StO_2_ was less accurate than with BRUNO due to the presence of a negative offset. Secondary analysis of the data with the use of an h calculated from the optical properties of the brain layer did decrease the error of StO_2_ accuracy by 75%, highlighting the importance of the prior information about scattering. These findings agree with the results published by Deepak Veesa and Dehghani [41], who used NIRFAST to assess the impact of oxygenation changes in the brain with a constant ECT oxygenation on the accuracy of SRS. While brain StO_2_ recovered with SRS was burdened with a negative offset, changing the algorithm to account for scattering without the need for h improved the accuracy of estimating StO_2_ in the brain.

The dynamic range of SRS StO_2_ was limited in the phantom measurements. The cause is not exactly known; a contributing factor must have been the absorption by water in the phantom; the concentration of water was much higher than in biological tissue. It is likely that the biggest disagreements between MAESTROS and BRUNO StO_2_ occurred in the low oxygenation range. The accuracy of oxygenation measurements at such low values is however not crucial for clinical care as they are outside of physiologically meaningful values. BRUNO showed best agreement with MAESTROS during oxygenation and deoxygenation changes in the range from ∼ 95% to 40%, which is more crucial for clinical work.

BF responded to increases to oxygenation with a delay and detected desaturations earlier than MAESTROS, it is likely that it is more responsive to HHb concentration than HbO_2_ concentration. This is not surprising given the more distinct spectral features of HHb, and, as BF only utilised one detector at 30 mm SDS, the signal-to-noise ratio of the reflectance spectrum was lower than in the attenuation slope measurement.

The broadband wavelength range used in BRUNO was limited by the spectral range of CYRIL. The performance of the algorithm could be improved by an optimization of wavelengths, perhaps extending the range to capture HbO_2_ features at 670 nm. The effect of wavelength selection in our broadband measurements is evident when comparing the results obtained with BF in other literature; the error of recovering StO_2_ in Monte Carlo simulated data [28] was approximately four times smaller than the error of StO_2_ recovery in model 1, Fig. 3. This reduction of accuracy is likely because we used a different wavelength range than suggested by the authors of BF, who performed the fitting over a wavelength range from 680 nm to 850 nm [29]. It is possible that the exclusion of the shorter wavelength range below 710 nm led to an underestimation of c_HbO2_, decreasing the accuracy of BF in NIRFAST model 1 and in the phantom measurement at maximal oxygenation.

The use of broadband spectra in BRUNO opens the possibility of adding further chromophores to the analysis, such as cytochrome-c-oxidase. However, such additions to the algorithm need to be validated using appropriate simulations, phantoms and *in vivo* models.

A NIRS measurement collected in an infant with HIE was used to demonstrate the use of a data quality assessment before data analysis. Including such a step is an advantage – any oximetry algorithm can analyse an arbitrary spectrum (e.g. with poor signal, without any spectral features). In a real-time measurement where only the resulting StO_2_ is displayed, measurement errors can go unnoticed if the StO_2_ lies in a normal range. By including a data quality assessment step which is not only based on the closeness of the fit but also includes an assumption about the shape of the spectra, we are able to automatically highlight data points which might not lead to the correct StO_2_.

The scoring system was developed based on empirical observations of the closeness of the BRUNO fit to a sample of data with varying quality. The initial visual assessment was guided by how distinct spectral features were, this concept was then translated into the automated data assessment evaluation. The two wavelength regions used for the calculation of residuals, 750–770 nm and 825–840 nm, were selected because they cover the most distinct spectral features of HHb and water. Visual inspection of BRUNO fits showed that the high quality collected data matched the model well in those regions, while the closeness of fit was worse for lower quality data.

Due to the empirical nature of the data assessment process, we suggest that data are not automatically excluded from the analysis based on the score; it should be used for guidance. The used wavelength ranges can also be updated depending on the expected spectral features. The data quality assessment step will be incorporated in CYRIL software when it is updated to use BRUNO real-time; the computational burden of BRUNO was higher than SRS, but the duration of the analysis was compatible with real-time measurements.

One data set was excluded from the analysis based on high scores. If the quality of the data was not assessed with the scoring system and the data was analysed, the output would not necessarily be alarming. For example, analysing the excluded data set with SRS yielded a mean StO_2_ of 80%, which is within ranges reported in infants with HIE [54].

StO_2_ collected on the left side of the head (data with low evaluation score) with all algorithms was within ranges expected in infants with mild HIE on day 3 of life [1,55,56]. A large intrasubject variation of StO_2_ in both HIE group and control group was reported by Dehaes et al. [54], with most values in the range of 60–80% at 75 hours of age. Although the baseline StO_2_ measured with SRS was higher than with BRUNO and BF, it does not give an unusually high value and would not stand out. However, as an overestimation of StO_2_ compared to BRUNO and BF was already observed in NIRFAST model 3, it is possible that SRS measured a slightly higher baseline due to the inability to account for water absorption and instead increasing c_HbO2_ as seen in NIRFAST model 3.

One of the limitations of this work is the small sample size of neonatal data used; the results presented are not meant as a performance assessment of any method. Instead, the aim was to highlight the difference in StO_2_ induced by algorithm selection and demonstrate the application of BRUNO in neonatal data. Compared to BF and SRS, BRUNO recovered StO_2_ with the highest accuracy, dynamic response and with minimal ECT signal contamination.

Further work on BRUNO is focused on the application in human subjects, particularly neonates. Measurements on a larger cohort of subjects will allow for the measurement of the algorithm’s precision. Additionally, performing measurements with inhomogeneous phantoms and could provide further insight into the impact of extracerebral layers on the accuracy of the algorithms. Such information is crucial for the application of BRUNO in adult subjects. We expect the algorithm to be translatable to adults, but the implementation will require extensive testing considering the increased ECT layer thickness compared to neonates, and the potential use of increased SDS. Additionally, work with other NIRS instruments is required to obtain a more general comparison of algorithm performance.
